# Relationship between Sedentariness and Moderate-to-Vigorous Physical Activity in Youth: A Multivariate Multilevel Study

**DOI:** 10.3390/ijerph14020148

**Published:** 2017-02-04

**Authors:** Thayse Natacha Gomes, Donald Hedeker, Fernanda Karina dos Santos, Michele Souza, Daniel Santos, Sara Pereira, Peter T. Katzmarzyk, José Maia

**Affiliations:** 1 CIFI2D, Kinanthropometry Lab, Faculty of Sport, University of Porto, Rua Dr. Plácido Costa, 91, 4200-450 Porto, Portugal; d.monteiro.santos13@gmail.com (D.S.); sara.s.p@hotmail.com (S.P.); jmaia@fade.up.pt (J.M.); 2 Department of Public Health Sciences, University of Chicago, 5841 South Maryland Ave MC2000, Chicago, IL 6063-1447, USA; DHedeker@health.bsd.uchicago.edu; 3 Department of Physical Education, Federal University of Viçosa (UFV), Avenida Peter Henry Rolfs, s/n, Campus Universitário, Viçosa, MG 36570-900, Brazil; fernandak.santos@hotmail.com; 4 Centre of Sports, Federal University of Santa Catarina (UFSC), Avenida César Seara-Carvoeira, Campus Universitário Trindade, Florianópolis, SC 88040-900, Brazil; mcsouza85@hotmail.com; 5 Pennington Biomedical Research Center, Louisiana State University, 6400 Perkins Rd., Baton Rouge, LA 70808-4124, USA; Peter.Katzmarzyk@pbrc.edu

**Keywords:** moderate-to-vigorous physical activity, sedentariness, school, multivariate multilevel analysis

## Abstract

This study aimed to jointly analyse moderate-to-vigorous physical activity (MVPA) and sedentariness, and their correlates, in children within their school contexts, using a multivariate multilevel approach. The sample comprises 499 Portuguese children (284 girls) from 23 schools. MVPA and sedentary time were estimated by accelerometer. A set of predictor variables from both child and school levels was tested. Overall, schools explained a small amount of the total variance in both MVPA (5.6%) and sedentariness (3.2%), and a correlation coefficient of −0.45 (*p* < 0.05) was found between MVPA and sedentariness at the child level. Number of siblings and socioeconomic status (SES) were significantly associated with both sedentariness (SES: *β* = 2.372 ± 1.183; siblings: *β* = −8.127 ± 2.759) and MPVA (SES: *β* = −1.535 ± 0.421; siblings: *β* = 2.822 ± 0.977), but with opposite signs. Body Mass Index (BMI) (*β* = −4.804 ± 1.898) and sex (male) (*β* = 21.561 ± 3.496) were only associated with MVPA. None of the school correlates were statistically significant in their joint effects to simultaneously explain sedentariness and MVPA. These results suggest that although MVPA and sedentariness may be different constructs, they are correlated and this should be taken into account when designing strategies to reduce children’s sedentariness and increase their MVPA. In addition, the small effect of the school context on this relationship highlights the important roles of child and family characteristics.

## 1. Introduction

It is now widely accepted that moderate-to-vigorous physical activity (MVPA) positively affects child and adolescent health [1]. In contrast, sedentariness is considered a potential risk factor in youth, as it is linked to the increased prevalence of overweight/obesity [2] and metabolic risk factors [1].

Although physical activity and sedentariness are sometimes viewed as opposite sides of the same coin [3], supporting the “displacement hypothesis” which assumes that inactive behaviours may replace more active ones [4,5], recent studies suggest that sedentariness is an independent construct from that of physical activity [4,6,7]. Studies have reported similar [2] and different correlates [8] of these two constructs, with distinct effect sizes and direction [7,9]. Further, these two behaviours can coexist [3], reinforcing the idea that the relationship between physical activity and sedentariness is far from being clear, with previous studies reporting conflicting results [4,9,10].

Both sedentariness and physical activity are linked with a wide array of biological, social, behavioural, and environmental correlates [2,8]. In this context, it has been suggested that sex [2], weight status [2], maturity [11], socioeconomic status (SES) [7,8], siblings’ influence [12], and sleep time [13] are related to time spent in these behaviours. Although schools are mainly viewed as delivering educational programs, taking into account the ecologic models of behaviours [14], the school environment may also provide opportunities for children to be physically active and to avoid extended sedentary periods through environments that may include playground areas, sports equipment and sporting facilities, recess periods, lunch breaks, and physical education classes [14,15,16]. Taken together, both child-level and school characteristics may explain a proportion of the variance in physical activity and sedentariness at the population level. Therefore, the aim of this study was to use a multivariate multilevel approach [17] to jointly analyse MVPA and sedentariness, investigating their relationship as well as their correlates in children within their school contexts.

## 2. Materials and Methods

### 2.1. Sample

The sample of the present study comes from the International Study of Childhood Obesity, Lifestyle and the Environment (ISCOLE) [18].

A total of 777 Portuguese children, aged 9–11 years, from 23 schools from the North of Portugal, were enrolled in the ISCOLE project. In each school, after the project was approved by the physical education department, school principal and parental council, all 5th grade students were invited to take part in ISCOLE, and those that were aged 9–11 years were eligible to participate after written informed consent was obtained from parents or legal guardians. From those, approximately 30 to 40 children were randomly selected per school (50% of each sex), and the response rate was 95.7%. After the inclusion criteria (children with valid accelerometer data and with no missing information on all other variables used in this study), the final sample comprised 499 children (284 girls).

The study protocol was approved by the University of Porto ethics committee (Protocol number 04/CEUP/2011), as well as by each school’s directorate councils.

### 2.2. Dependent Variable

Actigraph GT3X+ accelerometers (ActiGraph, Pensacola, FL, USA) were used to monitor MVPA and sedentary time. Children were asked to wear the accelerometer at their waist on an elasticized belt placed on the right mid-axillary line 24 h/day, for at least seven days, including two weekend days. To be eligible for this study, children had to have at least four days (from which at least one of them was a weekend day) with a minimum of 10 h of awake wear time per day. Accelerometer information was divided into daytime activities and nocturnal sleep time using an automated algorithm [19,20]. Non-wear time during the awake period was defined as any sequence of at least 20 consecutive minutes of zero activity counts [21].

Using cut-points advocated by Evenson et al. [22], different intensities of physical activity were determined. For the present study, mean MVPA and mean sedentary time were used, which were defined as being greater than or equal to 574 activity counts and less than or equal to 25 activity counts using 15 s epochs, respectively.

Additionally, and with the purpose to improve preciseness in parameter estimates and also to balance MVPA and sedentariness time in each child, their accelerometer wear time was considered in our statistical models.

### 2.3. Predictor Variables

#### 2.3.1. Child Level

##### Anthropometry

Stature, sitting height, and weight were measured according to standardized ISCOLE procedures and instrumentation [18]. Each child was measured twice with a permissible range between each measure and its replica of 0.5 cm for height and sitting height and 0.5 kg for weight; a third measurement was taken if the difference was outside the range. The mean value of each variable was used in all analysis.

The Body Mass Index (BMI) was computed with the standard formula (weight (kg)/height (m)^2^), and using the World Health Organization (WHO) [23] cut-points, children were classified as normal weight or overweight/obese.

##### Biological Maturation

Using information on sex, age, and physical growth characteristics (sitting height, leg length, stature, and body mass), an estimate of biological maturity was computed using the maturity offset method [24]. This method uses specific regression equations for boys and girls and estimates, in decimal years, the timing to peak height velocity (PHV) occurrence. A positive maturity offset expresses the number of years a child is beyond PHV, while a negative maturity offset means the number of years a child is before the PHV; a value of zero indicates that a child is experiencing his/her PHV.

##### Sleep Time

Using the accelerometer data with 60 s epochs, the nocturnal sleep duration for each participant was determined using a novel and fully-automated algorithm specifically developed for use in ISCOLE and epidemiological studies employing a 24-h waist-worn accelerometer protocol in children [19,20]. This algorithm produces more precise estimates of sleep duration, since it captures total sleep time from sleep onset to the end of sleep, including all epochs and wakefulness after onset. The mean sleep time across all days was used in the analysis.

##### Seasonality

Using information derived from the accelerometer (the period each child was assessed), children were classified as being monitored in Autumn (23 September to 20 December), Winter (21 December to 21 March), or Spring (22 March to 20 June), taking account the school academic year that comprises the period from September to June. Data were collected from September 2011 to January 2013.

##### Family Characteristics

Basic demographic characteristics were obtained via questionnaire, completed by parents or legal guardians (ISCOLE Demographic and Family Health Questionnaire [18]), which also provides information regarding ethnicity, family health, and socioeconomic factors. For the present study, we only used information about family SES and number of siblings. Socioeconomic status was determined by asking parents about the family annual income, and the answer was split into eight categories, ranging from <€6000 to ≥€42,000. For data analysis, these categories were centred at category 4. Parents were also asked about family size, informing the number of siblings the child enrolled in the project has.

#### 2.3.2. School Level

Information about the school environment was obtained via a questionnaire (ISCOLE School Environment Questionnaire [18]) completed by the physical education teacher or school principal. For the present study, the following school environment factors were considered: the school size (defined according to the number of students); the percentage of students participating in school sports or physical activity clubs; students’ access to outdoor facilities outside of school hours (0 = no; 1 = yes); and students’ access to playground equipment during school hours (0 = no; 1 = yes).

### 2.4. Data Analysis

**Figure 1:**
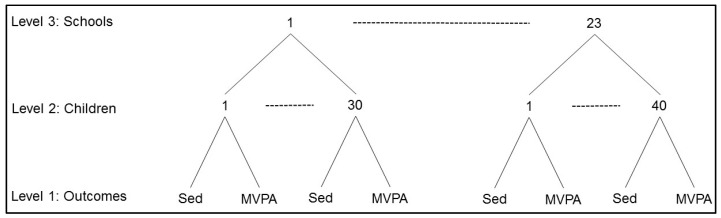
Multivariate multilevel structure of outcome variables (sedentary time (Sed) and moderate-to-vigorous physical activity (MVPA)) at level 1, nested within children at level 2, nested within schools at level 3.

Exploratory analysis, descriptive statistics, and *t*-tests to compare differences between boys and girls were done using SPSS 21 (IBM, Armonk, NY, USA). For the primary analyses, multilevel analysis was used to account for the nesting of students within schools. Also, given our interest in jointly modelling the two student-level outcomes of sedentary time and MVPA, a multivariate approach was used. Snijders and Bosker [17] have described several advantages of the multivariate approach (i.e., analysing multiple dependent variables jointly): (1) the possibility to obtain conclusions about the correlations between the dependent variables (in our case sedentariness and MVPA), notably the extent to which the unexplained correlations depend on child-level traits and school context variables; (2) a statistical increase in efficiency for tests of specific association on any single dependent variable given the multivariate nature of the data structure; (3) the ability to test whether the effect of any exploratory variable is similar or different across the multiple dependent variables; (4) the ability to avoid the capitalization on chance due to systematic tests being carried out on single dependent variables [25]. Thus, given the multivariate and clustered structure of our data, as shown in Figure 1, we utilized a three-level model: the two dependent variables sedentariness and MVPA are at level-1, child predictors are at level-2, and school correlates are at level-3.

All analyses were done using SuperMix software v.1, which implements full maximum likelihood estimation; the explicit formulation of this type of model and estimation details are described elsewhere [17]. As has been previously advocated [17], we used a three-step approach with random subject and school intercepts. In step 1 (baseline model, Model 1) the variances and covariances were jointly estimated for sedentariness and MVPA. These estimates allow us to calculate two important pieces of information: (i) how much of the total variation in sedentariness and MVPA is explained at the child and school levels; (ii) what is the size and direction of the correlations between sedentariness and MVPA for children within schools, and between schools. In step 2 (Model 2) we included child-level predictors, and in step 3 (Model 3) we added school context variables. Final decisions about the best fitting model were made using likelihood-ratio (LR) tests comparing nested models of increasing complexity. Specifically, a more complex model fits better than a simpler one if the difference in their respective deviances (−2 log L values) is statistically significant by the LR test. This is done using a chi-square statistic with degrees of freedom (df) equal to the difference in estimated parameters between the two models. For ease of presentation, we display the results for Models 1 to 3 according to Snijders and Bosker [17].

## 3. Results

Table 1 and Table 2 present descriptive statistics (mean ± SD and percentage) for child- and school-level variables. There is a high frequency (44.1%) of overweight/obese children. On average, children are about two years from their estimated PHV, and girls are more mature than boys. Children sleep about 8 h·day^−1^, and wore the accelerometer (during awake periods) for about 15 h·day^−1^ (ranging from 13 h·day^−1^ to 18 h·day^−1^). Further, children have one sibling on average, and 49.5% live in a family with an annual family income below €12,000. Almost half of the children were evaluated in winter (46.3%).

More than 90% of the schools in the sample have children engaged in sports participation or physical activity clubs. About half of the schools allow the students to have access to sports equipment outside school hours, but only 8.7% of them allow their students to have access to playground equipment during school hours. The mean number of students per school is 782 ± 309, ranging from 239 to 1589.

The multivariate multilevel modelling results are presented in Table 3, Table 4 and Table 5. Model 1 (Table 3) is the starting point and shows that, on average, these 10-year-old children have 555.1 min per day of sedentariness, together with 55.7 min per day of MVPA. Schools explain a relatively small amount of the total variation of both sedentariness (3.2%) and MVPA (5.6%). The major portion of the variance in sedentariness (96.8%) and MVPA (94.4%) is at the child level. Further, at the child level (within-schools), the covariance between sedentariness and MVPA is significant (*σ*_CL_ = −530.51 ± 60.03, *p* < 0.05) which translates to a negative correlation coefficient within child as *ρ*_CL_ = −0.45.

Model 2 (Table 4) includes a set of child characteristics (BMI category, maturity offset, SES, number of siblings, sex, sleep time, accelerometer wear time, and seasonality). Number of siblings and SES were associated with both sedentariness and MPVA, but with opposite signs: children with higher SES are more sedentary (*β* = 2.372 ± 1.183, *p* = 0.04) and spend less time in MVPA (*β* = −1.535 ± 0.421, *p* < 0.001); as SES was coded from 1 to 8, each level of SES resulted in 2.4 more minutes of sedentariness and 1.5 less minutes of MVPA. In terms of siblings, each additional sibling results in about an 8 min decrease in sedentary time (*β* = −8.127 ± 2.759, *p* = 0.003) and nearly 3 more minutes of MVPA (*β* = 2.822 ± 0.977, *p* = 0.003). BMI and sex were only associated with MVPA such that overweight/obese children are less physically active than their normal weight peers by nearly 5 min (*β* = −4.804 ± 1.898, *p* = 0.01), and boys are more physically active than girls by over 20 min (*β* = 21.561 ± 3.496, *p* < 0.001). Moreover, a significant effect of accelerometer wear time was observed for sedentariness (*β* = 0.424 ± 0.085, *p* < 0.001), and regarding seasonality, children tend to spend less time in MVPA during autumn (*β* = −7.745 ± 3.267, *p* = 0.018) and more time in sedentariness during winter (*β* = 15.336 ± 7.421, *p* = 0.039), compared to spring (our reference category). The child-level negative correlation (within schools) increased relative to the previous model ρ_CL_ = −0.54. This model fits better than the previous one given the reduction in deviance (∆ = 576.6851, 18 df, *p* < 0.001).

The final model (Model 3) included school level covariates (Table 5). None of the covariates were observed to be statistically significant in their joint association to simultaneously explain sedentariness and MVPA (*p* > 0.05). Further, the small non-significant reduction in deviance (∆ = 4.2873, 8 df, *p* = 0.112) indicates that the previous model (Model 2), being more parsimonious, is the preferred model.

## 4. Discussion

Using a multivariate multilevel approach, the present study aimed to jointly analyse MVPA and sedentariness as well as their correlates in Portuguese children within their school contexts. A relevant finding of the present study is the negative correlation between MVPA and sedentariness at the child level, suggesting that children with higher levels of MVPA tend to also have lower levels of sedentariness even after adjusting for a set of covariates at the child and school levels.

In the present study, a negative correlation between MVPA and sedentariness was found. The relationship between MVPA and sedentariness is not always clear, and although they have been seen as two different individual traits [4,6,7], the co-existence of both in children can occur, suggesting that highly physically active children can be less sedentary than their low physically active peers. A recent review [26], aiming to identify clustering patterns of diet, physical activity, and sedentary behaviour in children/adolescents, reported that both cluster patterns, high physical activity/high sedentary behaviour and high physical activity/low sedentary behaviour, are observed in youth, supporting previous studies [3,27] that account that physical activity and sedentariness can sometimes compete with each other and can sometimes coexist. Further, Marshall et al. [9] studied the interrelationship between sedentary behaviours and physical activity in youth aged 11–15 years, and reported a positive correlation between these traits. It was also found that, among boys, 40% of them self-reported more sedentary behaviour, and 94% of the more sedentary boys participated in double the recommended physical activity guidelines necessary for health. On the contrary, Tammelin et al. [10] reported a negative association between physical activity and TV viewing and computer use, where the highest proportion of physically inactive individuals were observed among those who watched TV for at least 4 h∙day^−1^ (14% in girls, 13% in boys). These authors also reported that those who spent at least 4 h∙day^−1^ watching TV (prevalence ratio: 1.5 for boys and 2.5 for girls) or using the computer/playing video games for more than 2 h∙day^−1^ (prevalence ratio: 1.4 for boys and 2.2 for girls) were more likely to be physically inactive than those who watched TV or used the computer/played video games for less than 1 h∙day^−1^, respectively. Using physical activity and sedentary pattern data from a sample of 10–11 year-old children, Jago et al. [28] identified three distinct clusters that were subjectively labelled as “high active/low sedentary”, “low active/moderate sedentary”, and “high active/high sedentary”, highlighting that the presence of one behaviour does not exclude the presence of the other. The negative correlation found in the present study can be related to the fact that, since the 24 h day is finite, children that spend higher amounts of time in MVPA or sedentariness have less available time to engage in the other behaviour. Further, this result suggests that both sedentariness and MVPA can be considered when strategy planning and program implementation are prepared to reduce health risks in children.

When MVPA and sedentariness are investigated as single/individual outcomes they sometimes share similar correlates. For example, SES and number of siblings were common correlates in our multivariate model, but this association was in opposite directions: children with higher SES are more sedentary and less physically active, while those with more siblings are less sedentary but more physically active. There seems to be no consensus about the association between SES and physical activity or sedentariness [7,8,29]. For example, Tandon et al. [29], studying children aged 6–11 years, reported that lower SES home environments provide more opportunities for sedentary behaviour and fewer for physical activity, while Atkin et al. [12] found an increase in sedentary time over one year among children (mean age: 10.2 years) with higher SES. The results for physical activity are similarly diverse. For example, Newton et al. [30] found that lower SES African American boys spend more time in MVPA compared to middle SES African American and lower SES Caucasian children. Among British adolescents aged 11–12 years, followed for five years, [31] no significant association between boys’ physical activity and SES was observed, but among girls those from lower SES were less active. Our findings indicate that children from higher income families tend to have greater sedentariness and lower MVPA. This can be related to the fact that these children are more likely to have more access to media entertainment (such as TV, computer, games etc.) for use during their leisure time, reducing available time to spend in physical activity and thus increasing time spent in sedentary activities.

Results of the present study showed that children with siblings tend to be more active and less sedentary, yet the role of siblings as a correlate of children’s physical activity and sedentary behaviour is not clear. For example, Tandon et al. [29] reported that children, on average, tend to spend more days per week watching TV/DVDs with siblings than participating in physical activity. However, it was also demonstrated that the presence of more children at home is highly related to more MVPA overall and at home, as well as more overall sedentariness at home but less screen time [32]. As to the results of our study, a possible explanation may be that those children with less sedentary/more active siblings tend to share such behaviours, becoming less sedentary/more active too.

The simultaneous modelling of MVPA and sedentariness revealed that, when their predictors were jointly analysed, sex, BMI, and autumn are only associated with MVPA, but not with sedentariness. Sex differences in both sedentariness and MVPA have been previously reported, where boys are generally more physically active and also more sedentary than girls, especially among adolescents; however, in children, results are not conclusive regarding the relationship between sex and sedentariness [8]. It can be argued that, on the one hand, during leisure time girls largely tend to get involved in more sedentary activities (e.g., reading, listening to music, socializing with peers), while boys tend to engage in more intense physical activities (e.g., sports or competitive games) [33]; on the other hand, boys tend to be more interested in TV and video games which may make them more sedentary [34]. Our results, however, found no significant sex differences for sedentariness when simultaneously analysed with MVPA, meaning that the relationship between sedentariness and sex may be different when sedentariness is analysed as a single outcome and when the analysis is done examining the co-occurrence of MVPA and sedentariness at the child level.

A significant relationship was observed between BMI and MVPA, but we did not find a significant association between BMI and sedentariness. Studies focusing on the association between physical activity and weight status in children generally report a negative relationship between excess weight and physical activity [2], probably because overweight/obese children have poorer motor skill proficiency [35], lower physical perceived competence, and lower peer acceptability in sports [36] than their normal weight peers, and this cluster of “factors” may contribute to their lower MVPA levels. Our results may suggest that probably the most relevant variable related to excess weight is physical activity, and that both normal weight and overweight children tend to spend similar amounts of time in sedentary activities.

In our study, it was found that children tend to spend less time in MVPA in autumn as compared to spring, and more time in sedentariness in winter. It has been suggested that weather conditions can influence physical activity and sedentary levels. However, results are not always concordant, with studies reporting a negative trend in physical activity levels from summer to winter [37], while others reporting the inverse, a decrease in physical activity in summer compared to winter [38]; and regarding sedentariness, there is no conclusive evidence about its seasonal variation [37]. It is possible that a colder climate, in association with a lower light exposure in autumn and winter, lead children to reduce their physical activity levels and increase their sedentary activities, since they may tend to reduce their involvement in outdoor physical activity [39].

The accelerometer wear time was positively associated with time spent in sedentariness in children. Aadland and Ylvisaker [40], studying adults, found a positive association between accelerometer wear time and absolute time spent in sedentariness, which was fully attenuated when sedentariness was reported as percentage values. The results found in the present study, using absolute values of sedentariness, can be related to the fact that the more time children wore the device, the more time in sedentary activities was registered. Therefore, in order to minimize this effect, we included wear time as a covariate in the models with the purpose to adjust for this effect.

At the school level, no statistically significant covariance/correlation was observed. This could potentially be related to the smaller sample size at the school level (*n* = 23 schools); however, the correlation coefficient suggests that MVPA and sedentariness tend to cluster. Although we were not able to identify any specific school-level covariate that could be related to the co-occurrence of these behaviours, results indicate that such covariates exist. In any case, our results highlight the role of biological and demographical characteristics in regulating the clustering of physical activity and sedentariness in children, as well as the relevance of the school environment, since schools explain 3.2% and 5.6% of sedentariness and MVPA variance, respectively.

This paper has several limitations which should be discussed. First, the cross-sectional design does not allow for causal interpretation of the results. Second, it is possible that if we had more schools we would probably be more likely to show that the correlation between these two traits, MVPA and sedentariness, was also significant at this level. Third, the sample comes from only one Portuguese region, and results cannot be generalized to other areas, notwithstanding the fact that, in data not shown here, overweight/obesity [41] and prevalence and SES distribution [42] compares with previous studies. There are also several strengths in this study that deserve to be mentioned: (1) the use of a multivariate multilevel analysis to identify the joint “determinants” of MVPA and sedentariness at individual and school-levels, as well as their variance and covariance; (2) the use of an objective method to estimate MVPA and sedentariness, and (3) the use of highly standardized methods for data collection.

## 5. Conclusions

In conclusion, this study showed that there is a negative correlation between MVPA and sedentariness (i.e., that although MVPA and sedentariness are two different traits that can occur in children, they are correlated). Some relevant covariates at the child-level were significantly associated with both MVPA and sedentariness, such as number of siblings, SES, and season; however, BMI and sex were only related to MVPA. The school environment explains a small amount of the total variation of both sedentariness (3.2%) and MVPA (5.6%), and none of the school-level covariates were statistically significant in explaining simultaneously the variation in sedentariness and MVPA; however, the correlation coefficient at child-level of −0.54 suggests that sedentariness and MVPA are correlated at the individual level. This correlation implies that these two behaviours are not independent, nor are they simply opposite sides of the same coin (i.e., a perfect inverse correlation). This is relevant information that should not be neglected when planning strategies to promote physical activity and reduce sedentariness in youth.

## Figures and Tables

**Table 1:** Descriptive statistics for variables at the child level (level 1). MVPA, moderate-to-vigorous physical activity; BMI, Body Mass Index; SES, socioeconomic status; PHV, peak height velocity.

Variables		Child-Level Variables (Mean ± SD or Percentage)		
		Boys (*n* = 215)	Girls (*n* = 284)	Total (*n* = 499)
MVPA (min·day^−1^)		67.81 ± 23.75 *	46.82 ± 14.77	55.86 ± 21.79
Sedentary time (min·day^−1^)		544.01 ± 60.98 *	563.70 ± 53.78	555.21 ± 57.76
BMI (kg·m^−2^)		19.24 ± 3.29	19.32 ± 3.37	19.28 ± 3.33
Maturity Offset (years to PHV)		−2.77 ± 0.42 *	−1.24 ± 0.51	−1.90 ± 0.90
Sleep time (h·day^−1^)		8.18 ± 0.86 *	8.34 ± 0.84	8.27 ± 0.85
Wear time (h·day^−1^)		15.32 ± 0.84 *	15.16 ± 0.71	15.23 ± 0.77
Number of siblings		0.97 ± 0.79	0.98 ± 0.86	0.97 ± 0.83
BMI (classification)				
	Normal weight	51.6%	59.2%	55.9%
	Overweight/obese	48.4%	40.8%	44.1%
SES				
	<€6000	14.9%	22.2%	19.0%
	€6000–€11,999	32.6%	28.9%	30.5%
	€12,000–€17,999	20.0%	17.3%	18.4%
	€18,000–€23,999	11.2%	9.5%	10.2%
	€24,000–€29,999	7.9%	7.4%	7.6%
	€30,000–€35,999	5.1%	5.6%	5.4%
	€36,000–€41,999	2.8%	3.9%	3.4%
	≥42,000	5.6%	5.3%	5.4%
Season				
	Spring	22.8%	20.4%	21.4%
	Autumn	34.0%	31.0%	32.3%
	Winter	43.3%	48.6%	46.3%

* *p* < 0.05.

**Table 2:** Descriptive statistics for variables at the school level (level 2) (*n* = 23).

School-Level Variables (Mean ± SD or Percentage)	
Number of students (mean ± SD)	782 ± 309
Students’ participation in sports or physical activity clubs	
Not available	4.3%
Less than 10%	4.3%
10%–24%	34.8%
25%–49%	13%
≥50%	43.5%
Students’ access to outdoor facilities outside of school hours	
No	47.8%
Yes	52.2%
Students’ access to playground equipment during school hour	
No	91.3%
Yes	8.7%

**Table 3:** Model 1 main results (parameter estimates, Standard Errors (SE), and deviance) for both sedentary time and MVPA.

	Sedentary Time		MVPA		Covariance	
	Estimate	SE	Estimate	SE	Estimate	SE
			***Fixed Effects***			
Intercept	555.100 *	3.379	55.708 *	1.446		
			***Random Effects***			
Between-schools (school level)						
Variance	107.828	76.613	26.266 *	14.082		
Covariance					−31.256	26.077
Correlation					−0.588	
Within-schools (children level)						
Variance	3219.162 **	208.477	446.218 **	28.908		
Covariance					−530.505 **	60.034
Correlation					−0.445	
Deviance		9827.1370				

** *p* < 0.05; * *p* < 0.10. School explained variance for Sed = (107.828/(107.828 + 3219.162)) = 3.2%; and for MVPA = (26.266/(26.266 + 446.218)) = 5.6%.

**Table 4:** Model 2 (parameter estimates, Standard Errors (SE), and deviance) including child level predictors for both sedentary time and MVPA.

	Sedentary Time		MVPA		Covariance	
	Estimate	SE	Estimate	SE	Estimate	SE
			***Fixed Effects***			
Intercept	633.154 **	39.364	51.709 **	13.982		
Overweight/obese	−4.754	5.368	−4.804 **	1.898		
Maturity offset (years to PHV)	8.551	5.610	0.277	1.984		
Socioeconomic status	2.372 **	1.183	−1.535 **	0.421		
Number of siblings	−8.127 **	2.759	2.822 **	0.977		
Sex	−10.858	9.907	21.561 **	3.496		
Sleep time (h·day^−1^)	−6.545	4.651	−0.232	1.645		
Wear time (h·day^−1^)	0.424 **	0.085	0.054 *	0.030		
Autumn	2.137	7.852	−7.745 **	3.267		
Winter	15.336 **	7.421	−5.087 *	3.071		
			***Random Effects***			
Between-schools (school level)						
Variance	67.326	54.296	18.299 *	9.777		
Covariance					−25.560	19.150
Correlation					−0.728	
Within-schools (child level)						
Variance	2494.156 **	161.496	308.409 **	19.980		
Covariance					−472.284 **	45.624
Correlation					−0.539	
Deviance	9450.4519					

** *p* < 0.05; * *p* < 0.10.

**Table 5:** Model 3 (parameter estimates, Standard Errors (SE), and deviance) including child and school level predictors for both sedentary time and MVPA.

	Sedentary Time		MVPA		Covariance	
	Estimate	SE	Estimate	SE	Estimate	SE
			***Fixed Effects***			
Intercept	647.894 **	40.752	46.560 **	14.851		
BMI categories	−5.033	5.357	−4.738 **	1.896		
Maturity offset (years to PHV)	8.465	5.623	0.306	1.985		
Socioeconomic status	2.564 **	1.191	−1.583 **	0.424		
Number of siblings	−8.255 **	2.769	2.851 **	0.979		
Sex	−10.846	9.931	21.579 **	3.499		
Sleep time (h·day^−1^)	−6.484	4.668	−0.272	1.648		
Wear time (h·day^−1^)	0.432 **	0.086	0.052 *	0.030		
Autumn	−3.439	9.459	−5.723	4.259		
Winter	14.492 **	6.899	−4.779	3.070		
Students’ involvement in physical activity or sports Clubs	−0.357	2.890	0.110	1.296		
Students’ access to outdoor facilities	−2.253	6.012	1.297	2.738		
Students’ access to playground equipment	12.657	9.392	−3.972	4.322		
School size	−0.014	0.010	0.005	0.005		
			***Random Effects***			
Between-schools (school level)						
Variance	25.753	41.375	15.461 *	8.908		
Covariance					−15.132	15.665
Correlation					−0.758	
Within-schools (child level)						
Variance	2504.747 **	162.086	308.288 **	19.970		
Covariance					−473.317 **	45.700
Correlation					−0.539	
Deviance		9446.1646				

** *p* < 0.05; * *p* < 0.10.
